# Compact circularly polarized truncated square ring slot antenna with suppressed higher resonances

**DOI:** 10.1371/journal.pone.0172162

**Published:** 2017-02-13

**Authors:** Mursyidul Idzam Sabran, Sharul Kamal Abdul Rahim, Chee Yen Leow, Ping Jack Soh, Beng Wah Chew, Guy A. E. Vandenbosch

**Affiliations:** 1 Wireless Communication Centre, Universiti Teknologi Malaysia, Skudai, Johor, Malaysia; 2 Advanced Communication Engineering (ACE) CoE, School of Computer & Communication Engineering, Universiti Malaysia Perlis, Arau, Perlis, Malaysia; 3 Intel Microelectronics, Halaman Kampung Jawa, Penang, Malaysia; 4 Department of Electrical Engineering, Katholieke Universiteit Leuven, Leuven, Belgium; Drexel University, UNITED STATES

## Abstract

This paper presents a compact circularly polarized (CP) antenna with an integrated higher order harmonic rejection filter. The proposed design operates within the ISM band of 2.32 GHz– 2.63 GHz and is suitable for example for wireless power transfer applications. Asymmetrical truncated edges on a square ring create a defected ground structure to excite the CP property, simultaneously realizing compactness. It offers a 50.5% reduced patch area compared to a conventional design. Novel stubs and slot shapes are integrated in the transmission line to reduce higher (up to the third) order harmonics. The proposed prototype yields a -10 dB reflection coefficient (S_11_) impedance bandwidth of 12.53%, a 3 dB axial ratio bandwidth of 3.27%, and a gain of 5.64 dBi. Measurements also show good agreement with simulations.

## Introduction

There is a growing demand for smaller and more flexible antennas with circular polarization, allowing the antennas to be installed with various orientations while keeping a constant link performance [1]. This is not only the case for communications, but also for wireless power transfer (WPT) where misalignments between transmitter and receiver may reduce the conversion efficiency [2]. Such technology can be capitalized by various wireless communication systems to provide alternative power to sensor nodes, modules and tags. Wireless power transfer (WPT) or Microwave Power Transfer (MPT) is an application used to transfer power wirelessly between two points. It also can be defined as radio frequency energy harvesting or transports and it preferred for running low power sensor [3]. At the transmitting side, power is converted to microwaves through a microwave generator before being transmitted through free space. It will then be received by a special device called a rectifying antenna (rectenna) and converted back to power. Several demonstrated examples of such system and it history are given in [3]. WPT usually involves combining the antenna with a low pass filter (LPF) and diodes to form a rectenna for the direct conversion of radio frequency (RF) energy to direct current (DC) [4]. However, as diodes are non-linear components, they may generate harmonics which will then re-radiate from the antenna. This consequently decreases the efficiency of the RF to DC conversion. Therefore, filters are required to block the generated harmonics. To realize a compact structure with higher RF to DC conversion, a good solution is to combine the antenna with a filter forming a structure called a filtenna and for the antenna to operate with circular polarization (CP). Due to this demand, many techniques have been proposed to simultaneously accomplish compactness, CP, and higher harmonic rejection.

Methods to achieve compactness include introducing stepped-impedances [5], incorporating shorting-pins between patch and ground [6], using dielectric resonators [7], employing slots [8], and loading fractal slots on the radiating element [9]. Another promising solution to increase compactness featuring higher harmonic rejection is by introducing Defected Ground Structures (DGS). The existing ground plane can either be integrated using dumbbell [10], partial ring [11], or fractal [12] structures. It reduces the phase velocity of the wave which leads to slow wave effects [12–13], and improves the antenna in terms of compactness [14–15] and harmonic suppression [16]. On the other hand, compactness and higher harmonic suppression can be achieved simultaneously by introducing unique shaped as radiating element such as circular sectors on the radiator [17]. However, simultaneous control of the resonance and rejection frequencies is difficult to achieve using these techniques. To solve this, integration of additional bandpass filtering elements in the form of a compact microstrip resonant cell (CMRC) [18], and a window band pass structure [19] were proposed. The addition of such structures onto the transmission line is an excellent approach to miniaturize filtennas. However, the antennas in [18–19] are linearly-polarized planar monopoles with a low front to back ratio (FBR). Due to the heavy scattering environments and the polarization randomness of the arriving signals, the low FBR and linear polarization will lower efficiency of these filtennas when fixed with its rear backed up onto the surfaces of walls and ceilings. Besides that, a good alignment of a linearly-polarized transmitter antenna relatively with the orientation of the receiver is required to ensure better signal reception.

Several techniques which combine size reduction, CP and harmonic rejection have been reported. For example, in [20], a CP annular slot ring antenna with rejection of the second and third harmonic is presented. In [21], a conventional circular patch antenna with two peripheral cuts and four-right angle slits embedded in the radiating element enables the harmonic rejection feature. In [22] an asymmetrical square slot is implemented in the patch to enable CP, with an extra band pass filter on its reverse side [23]. In [24], integration of an unbalanced circular slot on a circular CP patch enables its miniaturization and higher harmonic rejection. However, only the second harmonic is rejected using this method, requiring an additional filter between the antenna and rectifier for higher harmonic suppression (of up to 8 GHz). Another example is the rectenna system in [25], realised using a combination of separate structures, resulting in a more complex and bulky structure.

In this letter, an antenna with circular polarization is realized for the first time to the best knowledge of the authors, by implementing an unequally truncated square slot ring on its edges onto the antenna ground forming a DGS. This is done in combination with a proximity-coupled feed, therefore improving the compactness of the structure [26]. A ladder-shaped stub and meander U-shaped slot arrangement in the transmission line enabled the higher harmonic suppression, avoiding the need for additional filtering, while maintaining design simplicity and compactness [27]. The final result is a compact CP antenna with up to third order harmonic suppression feature and the widest bandwidth at 2.45 GHz in comparison to available literature, besides offering circular polarisation. Measurement and simulation results are presented in Section III. A conclusion is drawn in Section IV.

## Design geometry

In this section, first the CP antenna design is presented, followed by the filter design methodology. The filtenna is designed for implementation on an inexpensive FR-4 board, with a permittivity of 4.5, and a loss tangent of 0.019. Simulations and optimizations were performed using CST Microwave Studio.

### Compact circularly polarized antenna

**Fig 1:**
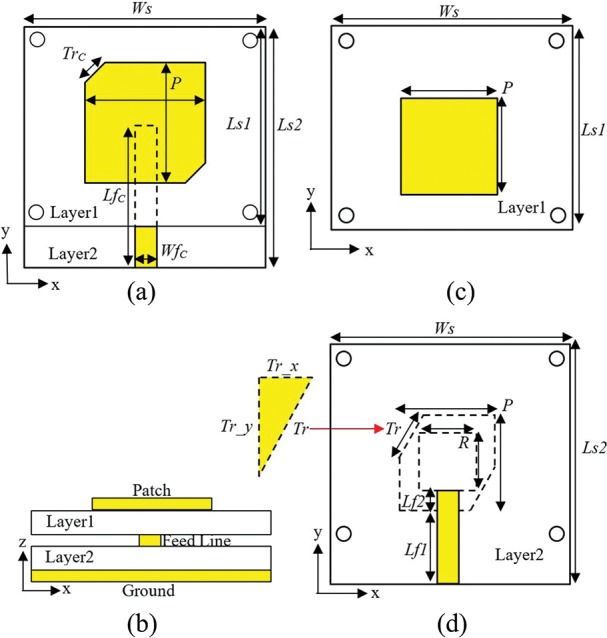
Geometry of the antenna structure. (a) conventional CP antenna (top view). (b) cross section. (c) compact CP antenna (top view of layer 1). (d) compact CP antenna (top view of layer 2).

The design starts with a classical design of a proximity coupled microstrip antenna [28], and ends with the new topology at the 2.45 GHz operating frequency. Both are shown in Fig 1. A non-contact, proximity coupled feed is introduced on an intermediate substrate layer backed by a full ground plane. The radiating elements are located on top of this layer containing the transmission line. Lengths of the bottom substrate (*Ls2* = 60 mm) and the top substrate (*Ls1* = 55 mm) are slightly different to enable the insertion of the 50 Ω SMA connector at the edge of the bottom substrate in practice. Four holes, each with 1.5 mm radius are added at the edges of both substrates to facilitate stacking. The initial length of the conventional square patch *P* is 0.23λ_o_ (27 mm) with 0.26λ_o_ (32 mm) length transmission line (*Lfc*) where λ_o_ is operating wavelength.

**Fig 2:**
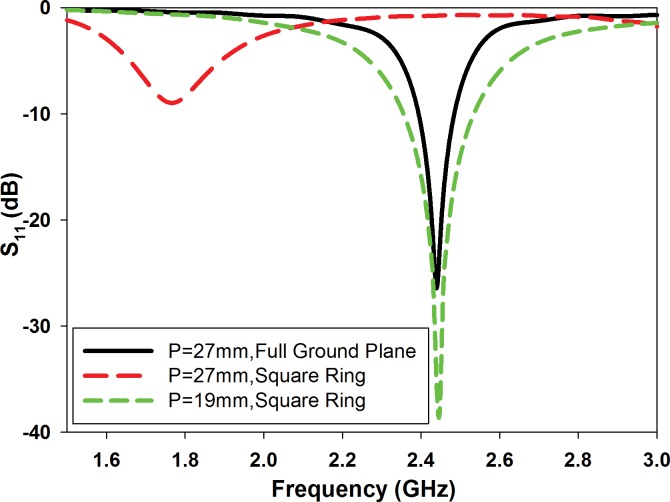
The reflection coefficients (S_11_) for the initial conventional and the compact CP antenna.

In order to realize a more compact antenna, the initial design is modified by adding 3 mm perimeter strip slot square ring onto the ground plane to form a DGS aligned underneath square patch. While the patch size is kept constant, the transmission line length (*Lf1*) underneath is reduced to 0.12λ_o_ (15 mm). The additional 0.024λ_o_ (3 mm) transmission line (*Lf2*) aligned underneath the square patch and its end is aligned with the border of the inner square ring (*R*), as shown in Fig 1D. This arrangement ensures that currents are excited around the square ring, which shifted the resonance to approximately 1.7 GHz with S_11_ = -9 dB, see Fig 2 (red dashed line). Miniaturization is enabled when the square ring slot is integrated as DGS introduced capacitance to the antenna [29]. This then lowers the antenna resonant frequency, and this principle is applied in optimizing the antenna size to result in a more compact final antenna at the desired frequency. As a counter-measure, *P* and *R* are then reduced to 19 mm and 16 mm to shift the resonance upwards towards to 2.45 GHz, see Fig 2 (green dashed line). Thus, a compact and miniatured patch design is obtained using the square ring DGS.

**Fig 3:**
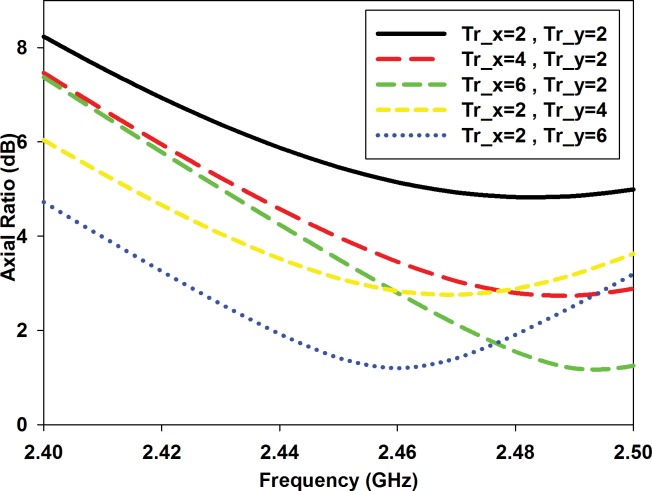
Parametric study of various values of Tr_x and Tr_y on AR.

Circular polarization is typically obtained when such antenna structure is excited using two orthogonal transverse modes with equal amplitudes which are 90° out of phase. Another traditional method is to use a single feed in combination with cuts at two opposite corners of the radiating patch (*Trc*), see Fig 1A. Alternately, modifying the square ring that forms the DGS is also a viable option, see Fig 1D. The ring is truncated asymmetrically with different values of *Tr_x* and *Tr_y* at the two opposite corners to excite CP. The implementation of an asymmetrically-truncated square ring as DGS as in Fig 1(D) to excite CP is quite unique in literature. It is observed that a larger *Tr_x* results in a higher minimum axial ratio (AR), whereas a larger parameter *Tr_y* lowers the frequency of the minimum axial ratio point, as shown in Fig 3.

**Fig 4:**
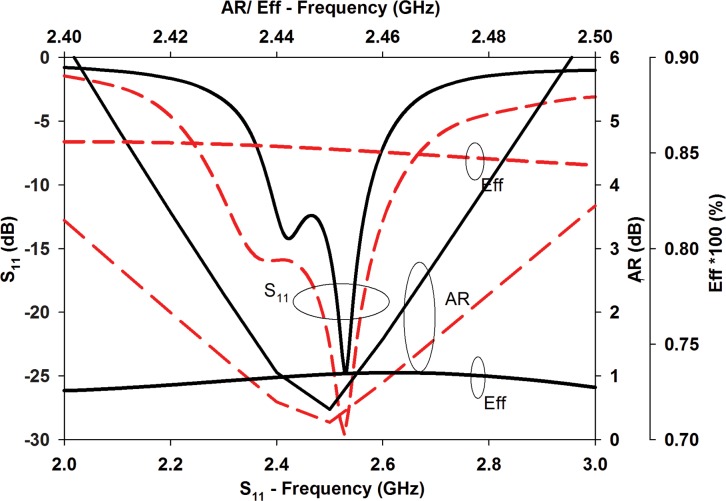
S_11_, AR, and radiation efficiency comparison between conventional antenna (black solid) and the proposed CP antenna (red-dashed).

Fig 4 compares the S_11_, AR and radiation efficiency (Eff) of the conventional and the proposed structure. The proposed structure provides a 50.5% reduction in terms of patch area compared to the conventional design. It also improves the 10 dB impedance bandwidth from 8.6% (2.38 GHz—2.58 GHz) to 13.8% (2.29 GHz– 2.63 GHz). Meanwhile, the 3 dB AR bandwidth improved from 2.45% (2.42 GHz– 2.48 GHz) to 3.27% (2.41 GHz–2.49 GHz) and the radiation efficiency increased from 73% (conventional topology) to 85% (proposed topology) at 2.45 GHz.

### Compact filter design

**Fig 5:**
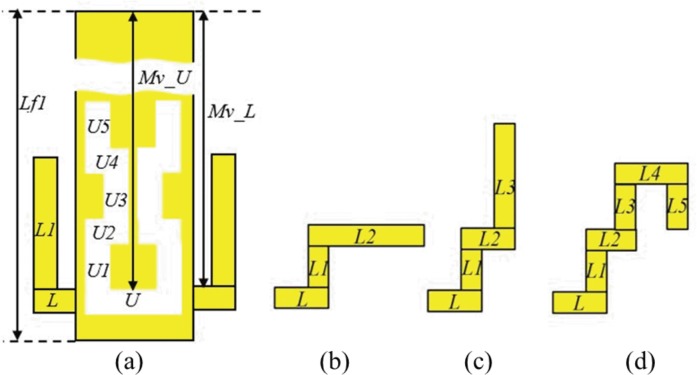
Evolution of the filter arrangement. (a) Meander U-slot and a pair of L-shaped stubs. (b) first evolution of the L-stub, Evo_1. (c) second evolution of the L-stub, Evo_2. (d) Final evolution of the L-stub.

**Fig 6:**
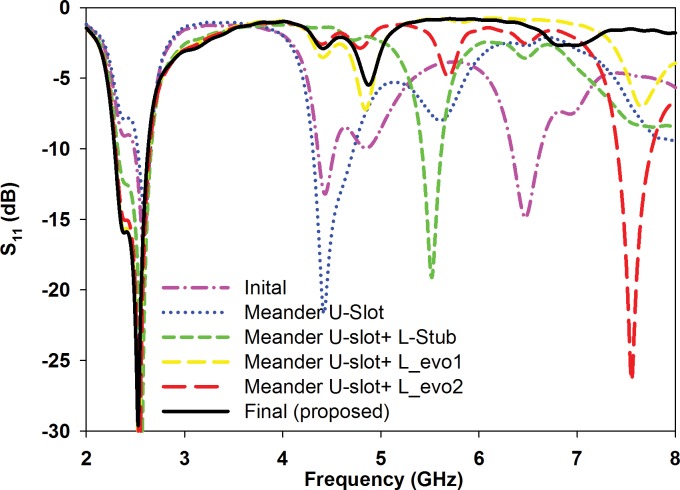
Reflection coefficient of the proposed antenna with different filter configurations.

In order to design a filter structure embedded within the antenna, several points need to be considered. It is important that the proposed technique does not affect the current flow on the patch and ground elements which will directly alter the reflection coefficient and axial ratio performance. Thus, one of the best solutions is to implement the filter onto the transmission line as shown in Fig 5. Novel stub and slots are defined to reduce resonance in the higher frequencies from 3 to 8 GHz. To ensure the structure’s compactness, the proposed filter is designed based on a complementary gap-coupled meander U-slot structure as illustrated in Fig 5A. The meandered slots are used to extend the electrical length for operation at the desired frequency without compromising compactness. The integration of the filter is initiated with the placement of the meander U-slot on the transmission line. This slot is based on the half-wavelength slot line filter concept and eliminates higher order resonant frequencies [30]. Total length of the meander U-slot is approximately 0.58λ_g_ at 6.48 GHz, where λ_g_ is the guided wavelength. The slot is 0.5 mm in width and is placed with a spacing of *Mv_U* = 0.73*Lf1* from the bottom edge of the patch. This immediately suppressed 6.48 GHz resonant frequency from the -14.79 dB (dash-dotted pink line) to -2.68 dB (dotted blue line) as shown in Fig 6.

**Fig 7:**
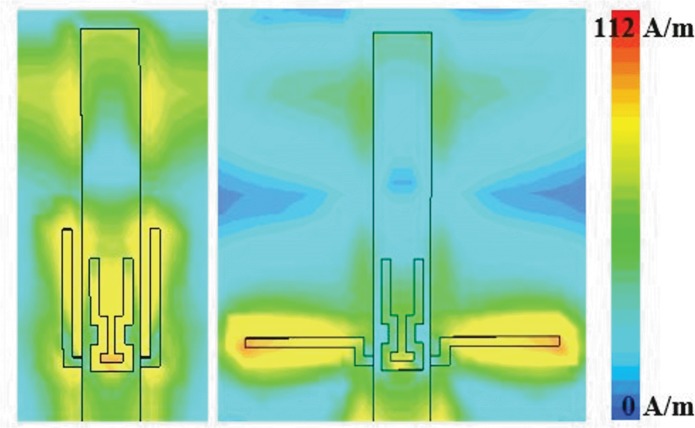
Simulated current distribution of different stub arrangement at 5.55 GHz. (a) L-stub. (b) L_evo1.

Next, a pair of complementary L-shaped stubs is added to the transmission line close to the meander U-slot. These stubs form parallel circuits with the antenna, and produce corresponding quarter guided wavelength stub resonances, in this way modifying the matching characteristics at a frequency that depends on the stub dimensions. Note that since in general mutual coupling of the meander and the stubs influence these resonances, they cannot be considered as just quarter wavelength stubs. The stub line width is 0.5 mm and the *L*, *L1* and *Mv_L* are optimized to be 0.75 mm, 7.5 mm and 0.73*Lf1 mm, respectively, in order to reduce the higher order resonance at 4.5 GHz, without changing drastically the matching at the fundamental resonance. The total length of the L-shaped stubs (*L*+*L1*) is 8.25 mm which is about 0.23λg at 4.5 GHz. The first higher resonance at 4.5 GHz is suppressed by increasing the S_11_ from -21.34 dB (dotted blue line) to -1.33 dB (dashed green line), see Fig 6. However, an additional resonance is then introduced at 5.55 GHz (dashed green line), see Fig 6, approximately between the original second and third harmonics. This is caused by the fact that the L-shaped stubs form a kind of matching network at this frequency, due to the tight mutual coupling with the transmission line. In Fig 7A it is clearly shown that the wave travelling on the transmission line is not stopped by the stubs.

To eliminate this unwanted higher resonance, the length *L1* is further modified by a 90° bend, creating the horizontal part with length *L2* = 0.23λg. This modification is labelled as *L_evo1* with *L* = 0.75 mm, *L1* = 1.5 mm, and *L2* = 6 mm in Fig 5B. This results in the elimination of the unwanted harmonic shown in Fig 6 (see the dashed yellow line). It is shown in Fig 7B that the effect of matching is eliminated. The wave does not continue any more along the transmission line. Next, the structure is further modified to form the configuration denoted as *L_evo2* as shown in Fig 5(C) to improve its compactness. It consists of elements *L* = 0.75 mm, *L1* = 1.5 mm, *L2* = 1.5 mm, and *L3* = 4.5 mm where the vertical *L3* element again generates another unwanted resonance at 7.6 GHz, see Fig 6 (dashed red line). Therefore, it can be concluded that any vertical stub placed in proximity of the transmission line may cause high mutual coupling and create additional unwanted resonances.

The final stub is shown in Fig 5D. The L-shaped stub is modified into a ladder structure mainly to avoid high mutual coupling and circumvent the generation of additional resonances when the stub located close to the transmission line. The meander U-slot is integrated with an additional bend to electrically lengthen it. This is to compensate for the upward shift in frequency caused by the ladder-shaped stub, effectively eliminating higher order harmonics while attaining the best performance at 2.45 GHz. The optimized filtenna dimensions are summarized in Table 1.

**Table 1:** Optimum parameter values of the proposed design (in mm).

Structure	Parameter / value (mm)			
**CP Antenna**	*Ws*	60	*P*	19
	*Ls1*	60	*R*	16
	*Ls2*	55	*Tr_x*	3
	*Lf1*	12	*Tr_y*	7
	*Lf2*	3		
**Filter**	**Meander U-Slot**		**Ladder Stub**	
	*Mv_U*	11	*Mv_L*	11
	*U*	2.20	*L*	0.75
	*U1*	0.60	*L1*	1.50
	*U2*	0.40	*L2*	1.50
	*U3*	1.50	*L3*	1.50
	*U4*	0.40	*L4*	1.50
	*U5*	3.25	*L5*	1.50

## Results and discussion

**Fig 8:**
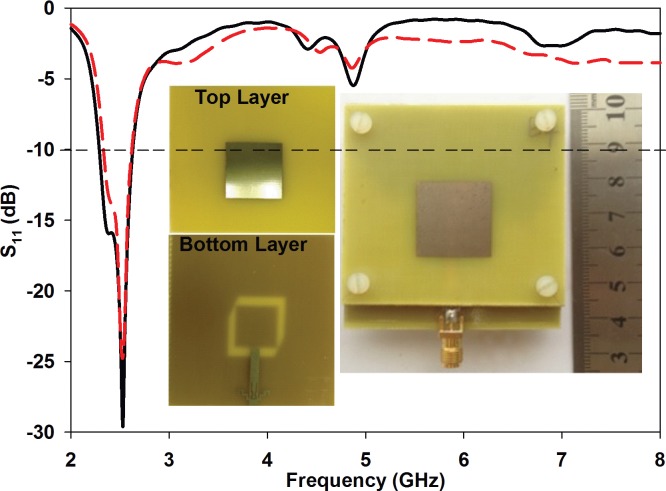
Simulated (solid line) and measured (dash line) S_11_ with photograph of fabricated antenna.

To validate the S_11_, axial ratio and radiation pattern experimentally, the proposed filtenna prototype was fabricated and measured. It can be observed that there is good agreement between simulated and measured reflection coefficients, see Fig 8. The fabricated filtenna operates between 2.32 and 2.63 GHz with a 10 dB impedance bandwidth of 310 MHz. The minimum S_11_ achieved within this range is -25 dB at 2.53 GHz. On the other hand, simulations featured about 400 MHz of bandwidth (2.23–2.63 GHz), with S_11_ of -30 dB. One of the main reasons for this difference is the difficulty in ensuring the accuracy of the gap between the top and bottom substrates during experiments. The simulation and measurement comparison is summarized in Table 2.

**Table 2:** Filtenna performance, simulated vs. measured.

Performances	Simulation		Measurement
**1^st^ resonant**	**Conventional**	**With DGS**	**With DGS**
**-10dB S**_**11**_ **bandwidth (MHz)**			
	(2380–2580)	(2290–2630)	(2320–2630)
	200	340	310
**Minimum S**_**11**_ **(dB) at frequency (GHz)**	-25.30 at 2.52	-29.53 at 2.52	-24.68 at 2.52
**3dB AR bandwidth (MHz)**			
	(2420–2480)	(2410–2490)	(2410–2490)
	60	80	80
	**Without filter**	**With filter**	**With filter**
**Higher resonant (GHz)**	**Minimum S**_**11**_ **(dB)**		
4.45	-12.93	-2.89	-2.33
5.50	-18.05	-0.84	-2.45
6.60	-14.63	-1.18	-2.92
7.35	-4.65	-2.01	-3.51

**Fig 9:**
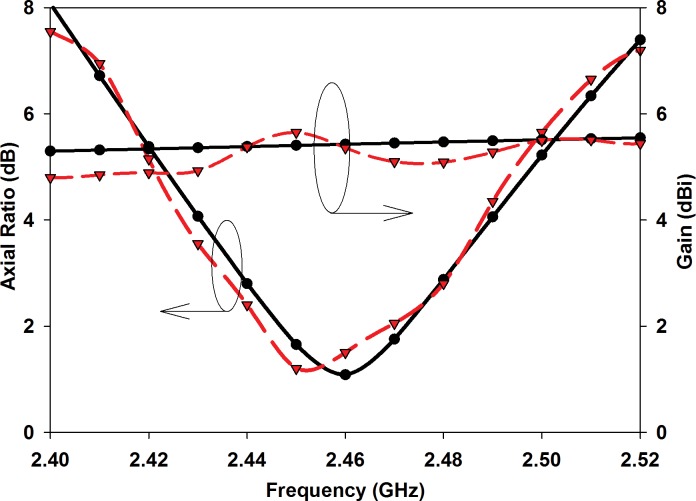
Simulated (solid line) and measured (dash line) gain and axial ratio.

Fig 9 compares the simulated and measured axial ratio and gain of the proposed structure. Simulated and measured results are in good agreement. The proposed filtenna has a measured gain in the +z-direction of 5.64 dBi at 2.45 GHz. The minimum axial ratio values are 0.27 dB at 2.45 GHz (simulated) and 1.32 dB at 2.41 GHz (measured). The simulated and measured 3 dB axial ratio bandwidths are 80 MHz or 3.27% (from 2.41 to 2.49 GHz). This axial ratio bandwidth is sufficient for WPT in the IEEE 802.11 wireless local area networks (WLAN) standard, widely used in indoor ambient environments.

**Fig 10:**
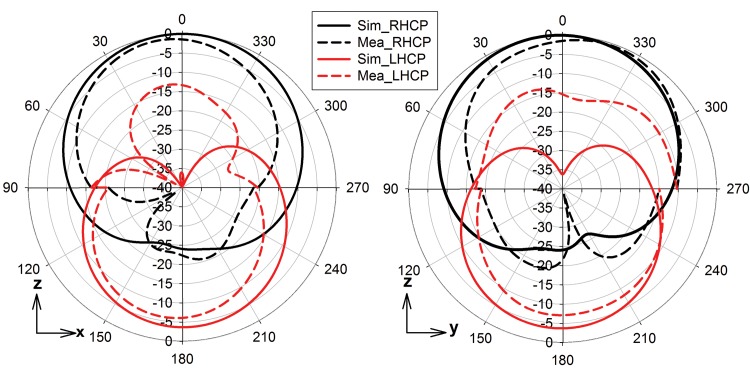
Radiation pattern at 2.45 GHz, simulated and measured. (a) XZ-plane. (b) YZ-plane.

A comparison of simulated and measured radiation patterns in the x-z (ϕ = 0°) and y-z (ϕ = 90°) planes is shown in Fig 10. They agree reasonably well. It is seen that the structure radiates right hand CP (RHCP) in the upper-half space. The 3-dB beamwidth in both planes is about 87°. The simulated and measured polarization ratios in both planes are larger than 15 dB in the boresight direction. However, left hand CP (LHCP) is better at the negative z-direction due to the effect of DGS-ring structure. Table 3 benchmarks the proposed filtenna with other circularly polarized antennas with a harmonic rejection mechanism, among which two operating at 5.8 GHz [22,23]. It can be observed that the proposed filtenna features a higher patch size reduction ratio, acceptable gain and reduces higher harmonics between 4 and 8 GHz compared to other reported 2.45 GHz microstrip filtennas.

**Table 3:** Comparison of compact circularly polarized antennas with harmonic rejection features.

Conventional	[20]	[21]	[22,23]	[24]	This work
**Patch shape**					
Square	Annular	Circular	Square	Circular	Square
**Centre frequency, f**_**c**_ **(GHz)**					
2.45	2.45	2.45	5.8	2.45	2.45
**Dielectric constant**					
4.5	3.38	4.4	2.2, 3.38	4.4	4.5
**Total thickness (mm)**					
3.2	1.524	1.6	4.8	2.5	3.2
**Patch length (mm)**					
27	43	32.2	14.8	31	19
0.22 λ_fc_	0.35 λ_fc_	0.26 λ_fc_	0.29 λ_fc_	0.29 λ_fc_	0.16 λ_fc_
**Patch area (mm**^**2**^**)**					
729	1452	814	219	682	361
0.048(λ_fc_)^2^	0.096(λ_fc_)^2^	0.052(λ_fc_)^2^	0.084(λ_fc_)^2^	0.047(λ_fc_)^2^	0.026(λ_fc_)^2^
**-10 dB S**_**11**_ **bandwidth, BW (MHz)**					
-	Not provided	137 MHz	630	137	310
**3 dB AR bandwidth, BW (MHz)**					
-	200	30	630	30	80
**Gain at centre frequency (dBi)**					
-	5.25	3.6	8	3.36	5.6
**Bandwidth of higher rejection (GHz)**					
	Not provided	(3–9)	(6–18)	(3–8)	(3–8)
**Bandwidth of non-rejection (GHz)**					
-	Not provided	(4.3–4.5)	Rejected all	(4.3–4.2)	Rejected all

## Conclusion

A compact CP microstrip antenna integrated with a higher harmonics rejection filter is presented in this investigation. The antenna is compact, with a size reduction of about 50.5% compared to a conventional design. CP is obtained with a square ring in the framework of a DGS. The ring enables both compactness as well as the CP property, through the asymmetrical truncated edges on its outer ring. A combination of slot and stubs on the transmission line provides adequate reduction of higher harmonics up to the third order. The proposed filtenna shows a good reflection coefficient performance, with a -10 dB impedance bandwidth of 310 MHz (2.32–2.63 GHz) in the ISM band. A directional radiation pattern is obtained.
